# Quantitative imaging methods for heterogeneous multi-component films†

**DOI:** 10.1039/d3sm01212c

**Published:** 2023-11-06

**Authors:** Ellard Hooiveld, Maarten Dols, Jasper van der Gucht, Joris Sprakel, Hanne M. van der Kooij

**Affiliations:** a Physical Chemistry and Soft Matter, Wageningen University & Research Stippeneng 4 6708 WE Wageningen The Netherlands hanne.vanderkooij@wur.nl; b Laboratory of Biochemistry, Wageningen University & Research Stippeneng 4 6708 WE Wageningen The Netherlands

## Abstract

The drying of multi-component dispersions is a common phenomenon in a variety of everyday applications, including coatings, inks, processed foods, and cosmetics. As the solvent evaporates, the different components may spontaneously segregate laterally and/or in depth, which can significantly impact the macroscopic properties of the dried film. To obtain a quantitative understanding of these processes, high-resolution analysis of segregation patterns is crucial. Yet, current state-of-the-art methods are limited to transparent, non-deformable labeled colloids, limiting their applicability. In this study, we employ three techniques that do not require customized samples, as their imaging contrast relies on intrinsic variations in the chemical nature of the constituent species: confocal Raman microscopy, cross-sectional Raman microscopy, and a combination of scanning electron microscopy and energy dispersive X-ray analysis (SEM–EDX). For broad accessibility, we offer a thorough guide to our experimental steps and data analysis methods. We benchmark the capabilities on a film that dries homogeneously at room temperature but exhibits distinct segregation features at elevated temperature, notably self-stratification, *i.e.*, autonomous layer formation, due to a colloidal size mismatch. Confocal Raman microscopy offers a direct means to visualize structures in three dimensions without pre-treatment, its accuracy diminishes deeper within the film, making cross-sectional Raman imaging and SEM–EDX better options. The latter is the most elaborate method, yet we show that it can reveal the most subtle and small-scale microseparation of the two components in the lateral direction. This comparative study assists researchers in choosing and applying the most suitable technique to quantify structure formation in dried multi-component films.

## Abstract 2

We describe and compare three label-free methods to quantitatively visualize the composition of complex coatings, elucidating segregation patterns such as component layering.

## Graphical abstract

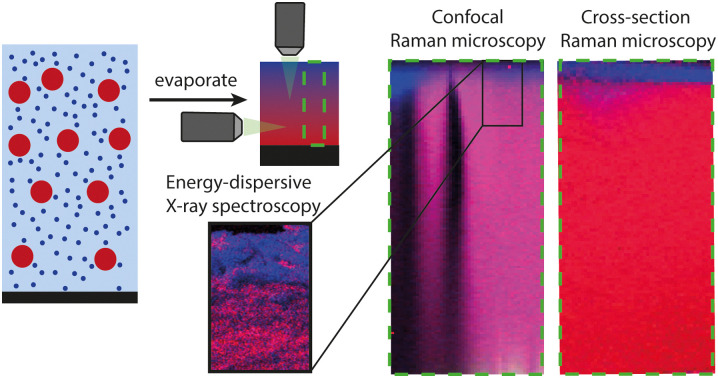

## 1 Introduction

The drying of colloidal dispersions occurs in a wide range of applications, including paints, glues, inks, and cosmetics. Depending on the components and the application, the final dry film can impart a diverse range of functionalities to the surface, such as adhesiveness,^1^ colour,^2^ antimicrobial qualities,^3^ mechanical robustness,^4^ electrical conductivity,^5^ water barrier properties,^6^ corrosion resistance, and more. These features strongly depend on the spatial distribution of the ingredients, which should in some cases be uniform, yet in other cases exhibit preferential partitioning to the top or to the substrate.

Waterborne coatings are a prototypical example of multi-component films whose performance and appearance rely heavily on the spatial arrangement of their building blocks.^7^ For example in nanocomposite films, a combination of soft and hard particles can be used to confer both strong adhesion and anti-fouling properties, achieved by partitioning of the adhesive particles to the substrate and hard particles to the air interface. By contrast, for optimal transparency a homogeneous distribution without segregation or aggregation of components is required. These examples illustrate the need to comprehend component distribution during drying, which is a diverse and complex process.

In the vertical direction, different pathways have been shown to cause spontaneous layer formation known as auto-stratification: (i) Sedimentation of the heavier species. Dense colloids tend to sink to the bottom of the film during evaporation, thus creating an enriched layer near the substrate.^8,9^ (ii) Capture at the evaporation interface. Slowly diffusing colloids may be swept up by the rapidly moving interface,^10^ or species may adsorb at the water–air interface to form a dense layer.^11^ (iii) Colloidal diffusiophoresis. In fast-drying dispersions that contain particles with different sizes, both species will initially accumulate at the evaporation interface, yet over time the large species will tend to move down the concentration gradient of the small species. This process manifests itself as a local depletion of large particles.^8,12–14^ (iv) Solvent flows. Near the end of drying, water suction through interstitial voids and pores can drag nanoparticles towards the top of the film, leading to an increased amount of those particles near the air interface.^3,15^

Which of these pathways happen(s), and to which extent, depend on many parameters such as the evaporation rate, volume fractions,^14,16^ particle sizes,^17,18^ particle size ratio,^14,16^ particle densities, particle interactions,^19^ particle stability,^20^ particle mobility,^21,22^ salt concentrations,^20^ and temperature.^23^ Navigating this vast parameter space is challenging and time-intensive, yet crucial for enabling the rational design of functionally graded coatings. Various techniques have been used to study the composition of films in the dry state. Atomic force microscopy (AFM) and attenuated total reflection Fourier transform-infrared spectroscopy (ATR-FTIR) can reveal excess of a species at the top surface by, respectively, scanning the topography^3,4,10,12,17,24,25^ or distinguishing chemical signatures.^2,26^

However, layers underneath the top remain obscured, making it impossible to obtain a complete picture of the component distribution throughout the film. Elastic recoil detection (ERD) can provide some depth-resolved information, but it requires deuterium labeling and only reaches up to 2 μm deep.^1^ Small-angle X-ray scattering (SAXS) offers access to the entire film depth but is limited to one dimension, leading to the averaging of lateral heterogeneities.^27–29^ Furthermore, it requires distinct particle boundaries, while most realistic coatings feature deformed and coalesced particles. Confocal laser scanning microscopy (CLSM) is a high-resolution imaging technique that allows real-time observations.^12,21,30^ However, it is mainly suitable for model systems, as at least one of the two particle types needs to be fluorescently labeled. In practical systems, labeling is not always feasible or may alter the particle chemistry.

Although the aforementioned methods have proven their merit, they are not generally applicable and lack combined depth and lateral resolution, which is greatly desired because most multi-component films exhibit inhomogeneities in multiple dimensions. One promising label-free, non-destructive, multi-dimensional imaging modality is confocal Raman microscopy (CRM). This technique relies on the analysis of Raman scattering of light to interrogate the vibrational modes of molecular bonds, enabling discrimination between components by identifying their unique chemical signatures. The confocal capability of CRM allows for optical sectioning, but recent work has greatly improved the depth resolution through cross-sectional imaging.^22,31^ Cross-sections have also shown potential for depth imaging of coatings using scanning electron microscopy (SEM), where contrast derives from morphological variations.^15,18,20,32^ In conjunction with energy dispersive X-ray analysis (EDX), which differentiates species *via* their elemental composition, this approach can even be applied to flat films from soft particles. Despite the great potential of these approaches, their capabilities for quantitative multi-dimensional imaging remain far from fully realised.

In this research, we present a comprehensive comparison of CRM, cross-sectional Raman microscopy (denoted by XRM), and a combination of SEM and EDX imaging. Our main objective is to guide the reader through the entire workflow, starting from the acquisition of raw data and leading to the accurate determination of the 3D composition of dried binary colloidal dispersions. To illustrate the process, we use a blend of hard silica nanoparticles and soft liquid latex particles commonly found in nanocomposite coatings. These species feature distinct vibrational modes and elements, making them well-suited for Raman and EDX imaging, respectively. We dry the blend at both room temperature and at 70 °C, giving rise to clearly distinguishable homogeneous and stratified distributions, respectively. We find that confocal Raman microscopy stands out as the most straightforward method, as it allows us to measure the chemical signature of both components throughout the entire depth of the film without any cross-section preparation. We leverage the full potential of this technique by converting Raman spectra into a quantitative 3D map of both species. Using XRM, we obtain very similar results, yet with greater depth resolution. Finally, the most involved methodology, SEM–EDX imaging, reveals additional types of lateral segregation within the film. This comparative study thus offers guidance on effective methods for analyzing heterogeneities in multi-component coatings, without the need of contrast agents.

## 2 Materials and methods

### 2.1 Materials

*n*-Butyl acrylate (BA) and methyl methacrylate (MMA) were purchased from TCI Europe. Sodium dodecyl sulphate (SDS) and 4,4′-azobis(4-cyanovaleric acid) (ACVA) were purchased from Sigma-Aldrich. 1 M KOH was purchased from Merck. Silica nanoparticles were provided by Nouryon. These particles have an average diameter of ∼16 nm and are surface-functionalized with a (3-glycidyloxypropyl)triethoxysilane (GPTES) coating, which provides steric stability and prevents aggregation during the drying process. All chemicals were used without any extra purification.

### 2.2 Latex synthesis

A 500 ml round-bottom (RB) flask was cleaned and etched by stirring a 1 M KOH solution for ∼1 h. Thereafter, the RB flask was flushed 3× with Milli-Q water. Subsequently, 100 mg SDS was dissolved in 150 ml Milli-Q water in the RB flask. To this solution 69 ml BA and 31 ml MMA were added to create a two-phase system. This ratio was chosen to create a latex with a *T*_g_ around −32 °C according to the Fox equation: 
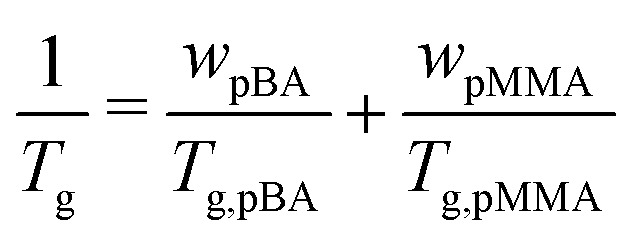
 in which *w*_pBA_ is the mass fraction of poly(*n*-butyl acrylate) (pBA), *T*_g,pBA_ = 209 K (−64 °C) is the glass transition temperature of pBA, *w*_pMMA_ is the mass fraction of poly(methyl methacrylate) (pMMA), and *T*_g,pMMA_ = 383 K (110 °C) is the glass transition temperature of pMMA. The RB flask was sealed with a rubber septum, and N_2_ gas was bubbled through the mixture for ∼15 min to deoxidize it. The flask was subsequently placed in an oil bath at 65 °C and tumbled at 50 rpm. After ∼15 min, the reaction was initiated by injecting 490 mg ACVA dissolved in 4.75 ml Milli-Q water and 5.25 ml 1 M NaOH. The mixture was further tumbled at ∼50 rpm for ∼24 h at 65 °C. Finally, the mixture was filtered through a glass wool filter to remove any coagulum.

### 2.3 Particle characterizations

The hydrodynamic diameters of the latex and silica particles were determined using dynamic light scattering (DLS). These measurements were performed on an ALV instrument equipped with an ALV-7002 external correlator and a 380 mW Cobolt Flamenco-300 laser operating at a wavelength of 660 nm. A 0.1 wt% disperison of both particle species was measured for 30 s in a polycarbonate capillary of 1.9 mm diameter (Enki SRL) at a detection angle of 90° and a temperature of 20 °C. These measurements were repeated 5×. The weight fractions of the latex and silica dispersions were determined gravimetrically to be 0.45 (≈43 v%) and 0.32 (≈15 v%), respectively.

### 2.4 Film drying

A mixture of 9.3 v% latex and 1.6 v% silica in Milli-Q water was dried on a silicon wafer at 20 °C or 70 °C in an enclosed environment to minimize the influence of convection on the drying process. 4 ml of mixture was spread out over ∼30 cm^2^ to provide an initial film height of ∼1 mm. For the cross-section measurements, the samples were immersed in liquid nitrogen several times. Hereafter some coating flakes came loose and were attached to an aluminum 90° SEM stub (Agar scientific, 12.7 mm diameter, 45/90° chamber, 9.5 mm pin) with thin carpet tape. For SEM–EDX measurements the same procedure was performed using electrically conductive and adhesive carbon tabs (EMS Washington USA).

### 2.5 CRM and XRM measurements

CRM and XRM analyses were performed on a WITec confocal Raman microscope using a 20× air objective (NA = 0.5). The sample was excited by a green 532 nm laser. The pinhole size was set at 50 μm through an optical fiber. The signal was collected on a CCD chip cooled to −60 °C. For the CRM measurements, a volume of 100 × 100 × 150 (*x* × *y* × *z*) μm^3^ was measured. A Raman spectrum was collected every 2 × 2 × 1 μm^3^, with an integration time of 0.15 s. These acquisitions took approximately 10 h in total. Note that the voxel dimensions can be tailored to the question at hand, with larger voxels reducing the measurement time but sacrificing resolution. Here, high resolution was prioritized to showcase the technique's potential. For the XRM measurements, the microscope was first focused on the cross-section of the film. Hereafter an area of 50 × 100–300 μm^2^ (depending on the film thickness) was scanned, covering at least the whole *z* direction. A Raman spectrum was collected every 1 × 1 μm^2^ with an integration time of 0.15 s. A calibration curve was created by mixing the latex and silica dispersions in different ratios to yield different volume fractions in the final dry films. These samples were dried at 4 °C in an enclosed chamber to ensure uniform drying, and measured just below the top surface at 5 random *xy* positions.

### 2.6 SEM–EDX measurements

First, the cross-sections were sputter-coated 2× at an angle of 45° with 5 nm iridium (MED 020 sputter-coater, Leica, Vienna, Austria). Between the two sputter-coatings, the sample was rotated laterally by 180° to ensure an even and uniform coating. This provided a conductive layer on top of the cross-sections. Hereafter, the samples were analyzed using a field emission electron microscope (Magellan 400, Thermo-Fischer/FEI, Eindhoven, the Netherlands) at 2 kV. Elemental analysis of silicon, carbon and oxygen was performed using an EDX spectrometer (Oxford Instruments, X-max, 80 mm^2^) attached to the Magellan 400. For EDX the samples were analyzed at 10 kV.

### 2.7 Raman and EDX data analysis

The raw Raman spectra were analyzed using a custom-written MATLAB script. In short, the signal of each Raman spectrum was first corrected for background scattering by subtracting the mean value of the intensities between *ν* = 1900–2300 cm^−1^. Then, the integral was taken of the intensities between *ν* = 425–470 cm^−1^ for the silica, between *ν* = 500–550 cm^−1^ for the silicon substrate, and between *ν* = 1700–1800 for the latex. For the cross-sections shown in Fig. 5, these signals were normalized by the total intensity of each spectrum. The analysis steps of the Raman data and the conversion to silica volume fractions (*ϕ*_SiO_2__) are further explained in the Results and discussion Section 3.1.

To determine the elemental percentages using EDX, specific positions within the sample were probed. These percentages were adjusted by excluding the traces of Al (mounting substrate), Na, K, and Ir (sputter-coated element). Additionally, the intensity of the Si signal was used in those specific areas to generate an intensity map representing the weight fraction of silicon (*w*_Si_). In both the Raman and EDX analyses, we assumed that *ϕ*_SiO_2__ + *ϕ*_latex_ = 1, thus disregarding the possible presence of trapped air in the sample.

## 3 Results and discussion

**Fig. 1:**
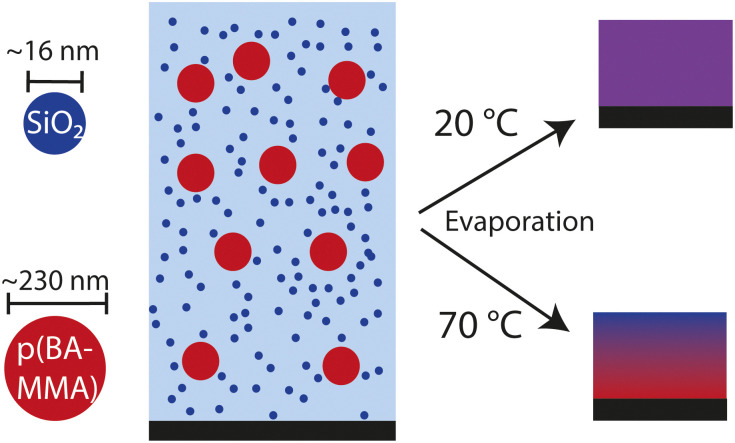
System of study. Drying a suspension of hard silica particles and soft polymer particles gives rise to a uniform film at low temperature but a stratified film at elevated temperature.

To benchmark and compare different label-free, quantitative, multi-dimensional imaging methods, we use a latex-silica coating system that dries homogeneously under ambient conditions but displays pronounced heterogeneities at elevated temperature. The used latex polymer, poly(BA-*co*-MMA), and silica particles have unique chemical identities that enable accurate spectral differentiation. Because depth-resolved optical analysis requires semi-transparency, the polymer particles must be sufficiently soft to coalesce and mitigate drying stresses, hence we select a polymer with glass transition temperature (*T*_g_) of approximately −32 °C. The initial dispersions contain 9.3 v% latex particles of ∼230 nm diameter and 1.6 v% silica particles of ∼16 nm diameter. The silica particles are sterically stabilised to prevent gelation or aggregation during the drying process.^20^ We dry this latex-silica blend as 1 mm thick films at 20 °C and 70 °C in an enclosed chamber. At elevated temperature, the evaporation rate is significantly higher than at low temperature, causing the silica and latex particles to accumulate at the drying interface. The resulting concentration gradient of the small silica particles subsequently leads to the displacement of the large latex particles to deeper regions in the film through diffusiophoresis.^8,12^ Consequently, we hypothesize that the final dry film will be stratified, in contrast to the film dried at 20 °C in which we expect a more homogeneous distribution (Fig. 1). These distinct scenarios provide an ideal platform for assessing the different microscopy methods.

### 3.1 Confocal Raman microscopy

**Fig. 2:**
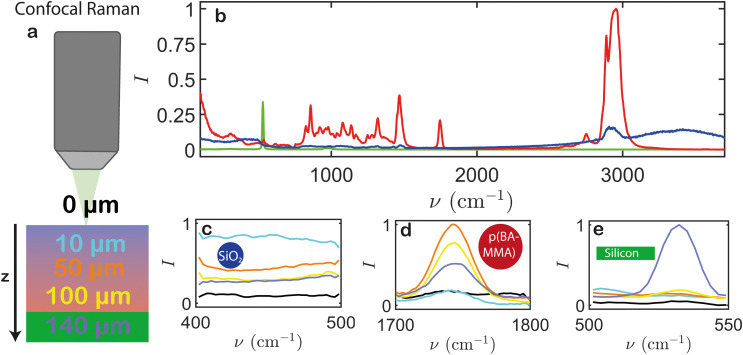
(a) Depth-resolved confocal Raman microscopy of a latex-silica dispersion dried at 70 °C. (b) Raman spectra of a latex-only film (red), pure silica particles (blue), and the silicon substrate (green). (c)–(e) Depth-dependent Raman spectra in wavenumber regions corresponding to (c) silica, (d) latex, and (e) silicon. The colour shading represents different measurements from the uppermost layer (air, 0 μm) to the lowermost layer (substrate, 140 μm) as shown in (a). This technique enables well-resolved discrimination of various substances across the entire film thickness.

First we examine the dried samples using CRM, which enables us to directly and non-invasively measure from the top to the bottom (Fig. 2a). We demonstrate the depth-resolving power on a sample dried at 70 °C, which we expect to stratify. To quantitatively interpret the acquired spectra, we need to compare them with the spectra of pure silica, pure latex, and the silicon substrate (Fig. 2b). In those spectra we select three wavenumber (*ν*) regions where only one of the three components shows a peak: *ν* = 400–500 cm^−1^ for silica, *ν* = 1700–1800 cm^−1^ for latex, and *ν* = 500–550 cm^−1^ for silicon. The silica particles also exhibit a broad peak at *ν* = 3100–3500 cm^−1^, yet this signal is attributed to the presence of OH groups and overlaps with residual water in the sample. We therefore exclude it from our analysis. Raman spectra of the three wavenumber regions for different *z*-slices are displayed in Fig. 2c–e, colour coded for the different depths. Because the top slice consists mostly of air, the corresponding signal is low in all cases. As we scan somewhat deeper, to 10 μm, we reach the uppermost slice within the sample, which shows a significant silica signal (Fig. 2c) yet a low latex signal (Fig. 2d), indicating enrichment of silica particles near the air interface. When we move deeper towards the core of the sample at 50 μm, the latex signal increases while the silica signal decreases. With further increasing depth to 100 μm, both the silica and latex signals are attenuated, due to the scattering of both excitation and emission light. This scattering originates from the elevated concentration of rigid silica nanoparticles near the film's surface, which hinders coalescence of the latex particles and leads to heterogeneous silica clusters and air-filled pores.^33^ Finally, focusing the laser on the substrate at 140 μm shows a strong enhancement of the silicon signal (Fig. 2e), while both the latex and silica signals continue to decrease. These results are in line with the expected stratification pattern at this drying temperature, shown schematically in Fig. 1 verifying that CRM allows differentiating multiple components throughout the full depth of the film.

**Fig. 3:**
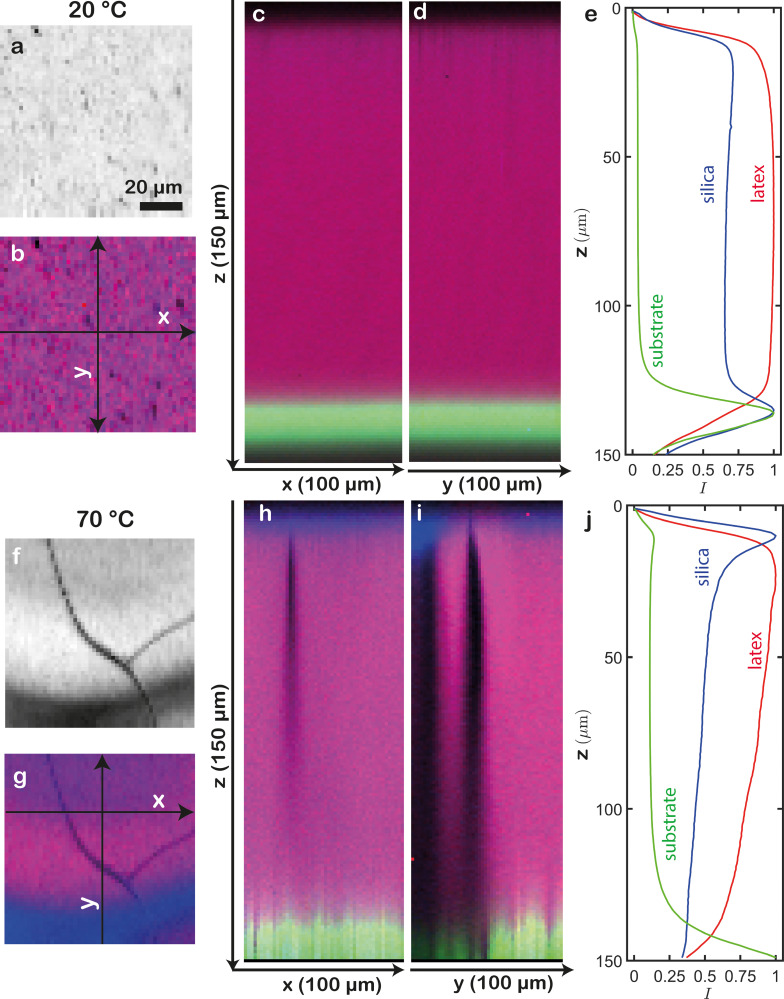
3D confocal Raman microscopy of a latex-silica film dried at (a)–(e) 20 °C and (f)–(j) 70 °C. 2D top views of (a), (f) the total integrated Raman signal and (b), (g) overlays of the latex signal (red) and silica signal (blue). 2D cross-section projections along the (c), (h) *xz* plane and (d), (i) *yz* plane, merging the latex, silica and substrate (green) signals. The corresponding projection axes (*x* and *y*) are indicated in (b) and (g). (c), (j) Average normalized silica, latex and substrate signals throughout the depth of the films. These CRM data highlight a homogeneous distribution in a film dried at 20 °C, yet strong compositional heterogeneities in a film dried at 70 °C.

To also examine the film in the lateral directions, we probe a volume of 100 × 100 × 150 μm^3^ (*x* × *y* × *z*), in which we collect a Raman spectrum every 2 × 2 × 1 μm^3^. We thus obtain a four-dimensional data set as a function of *x*, *y*, *z*, and *ν*. We subsequently integrate the Raman signals within the specific wavenumber regions corresponding to silica, latex, and substrate in each voxel. This allows us to semi-quantitatively reconstruct the film's composition distribution. To effectively visualize the spatial arrangement, we assign colour intensities to the different components that reflect the integrated local intensities. We choose to consistently colour-code latex in red, silica in blue, and the silicon substrate in green. Since 3D renderings are ambiguously interpretable, we first select 2D projections *i.e.* slices in different planes. For example, top views of the film dried at 20 °C are highly homogeneous (Fig. 3a and b). Both at the surface and in the deeper layers, the silica and latex signals largely overlap, yielding a uniform colour. The silicon substrate displays a sharp and strong peak at the bottom (Fig. 3c, d, Fig. S1a and Video S1, ESI†). These observations are further evidenced by averaging the three signals over the *xy* plane to create latex and silica profiles, which are constant throughout the depth, except near the boundaries (Fig. 3e). Most notably, a clear increase in silica signal is visible near the substrate–film interface, which does not represent accumulation of silica particles but rather arises from the silicon substrate. It is well-known that the silicon surface undergoes oxidation,^34^ resulting in a thin layer of silica that overlaps with the signal from the silica particles. Although this layer is only a few nm thick in reality, it appears to extend over >10 μm in our data. Part of this discrepancy is rooted in blurring of the signals from deeper layers, due to refractive index variations near the film–air interface. Another possible explanation is partial overlap between the silicon and silica signals, due to the proximity of their respective peaks in the Raman spectra. Interestingly, also near the film–air interface, a minor enrichment of silica is visible.

By contrast, the film dried at 70 °C exhibits various pronounced heterogeneities, such as cracks and uneven distributions of latex and silica (Fig. 3f–g). 2D projections along *y* and *x* show that the cracks extend into the deeper regions of the film, with the silica particles preferentially accumulating at the top (Fig. 3h–i, Fig. S1b and Video S2, ESI†). Averaging over the *xy* plane indeed shows a sharp silica peak near the film–air interface, indicative of stratification *i.e.* layering (Fig. 3j). We note that while the substrate signal is consistently flat in the film dried at 20 °C, consistent with reality, it appears to fluctuate strongly in the film dried at 70 °C (compare Fig. 3c, d and h, i). We attribute these fluctuations to the presence of cracks and reduced transparency in the sample, which cause light scattering and related optical artefacts. Most likely, the rapid drying at elevated temperature prevented sufficient time for complete latex particle deformation and coalescence, and consequent mitigation of drying stresses, ultimately leading to fracture. Additionally, the accumulation of silica at the top further introduces local refractive index heterogeneities.

**Fig. 4:**
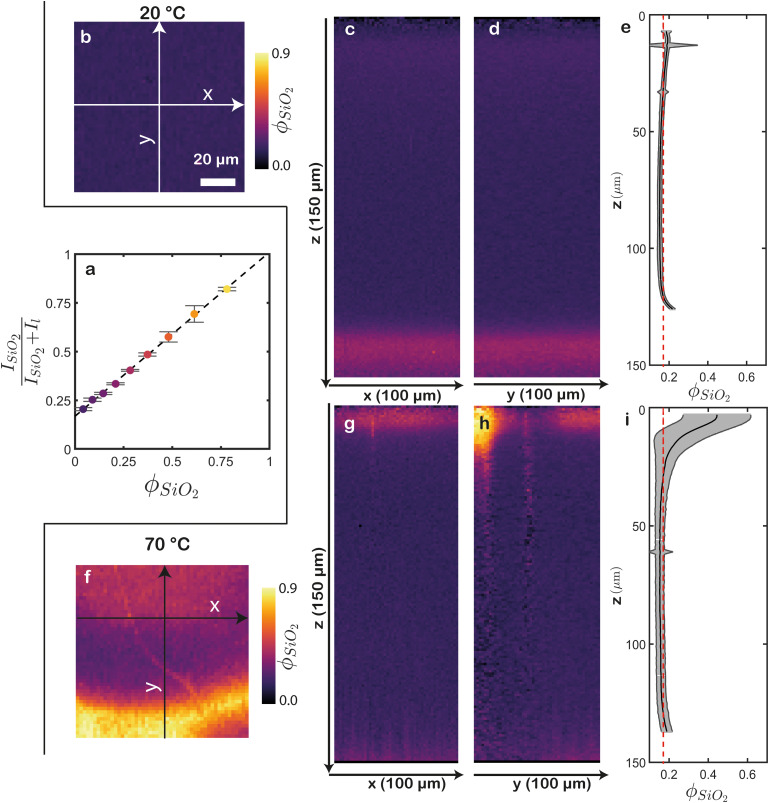
Conversion of Raman intensities to volume fractions. (a) Calibration curve of the normalized silica signal *versus* the silica volume fraction. The dashed line represents a linear least-squares fit of the data (*R*^2^ = 0.99). Error bars represent the standard deviation of five measurements at different positions. (b)–(i) *ϕ*_SiO_2__ maps of a film dried at (b)–(e) 20 °C and (f)–(i) 70 °C. (b), (f) Top views. Both images have the same scale. 2D cross-sections along (c), (g) *xz* and (d), (h) *yz*. The corresponding projection axes are indicated in (b) and (f). Close to the substrate, a small erroneous increase of silica content is visible, due to cross-talk between the substrate (silicon) and silica signals. (e), (i) Average silica volume fraction throughout the depth. Unreliable values near the air and substrate interfaces are cropped. The grey areas represent the standard deviations. The red dashed lines indicate the expected random *ϕ*_SiO_2__. This methodology enables precise quantification of compositional variations both within and between samples.

We realise the full quantitative potential of CRM by converting the Raman scattering intensities to volume fractions of the two components. To do so, we dry nine blends with different final volume fractions of latex and silica very slowly to prevent stratification of the components. We measure these films at the top and determine the integrals of the latex (*I*_l_) and silica (*I*_SiO_2__). We calculate the normalized ratio of these signals as 
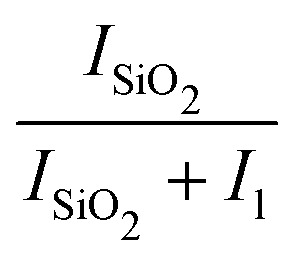
. In line with expectations, this ratio exhibits a clear linear relationship with the final volume fraction of silica (*ϕ*_SiO_2__) (Fig. 4a). This linear correlation can be used to directly convert raw Raman spectra to the silica volume fraction. Since this is a two-component system, the latex volume fraction is 1 − *ϕ*_SiO_2__. Doing this in a spatially resolved manner, *i.e.* voxel by voxel, yields 3D reconstructions of the silica and latex volume fractions. The resultant top and cross-section views corresponding to the previously described films are shown in Fig. 4. Examining the top of the film dried at 20 °C shows an expected uniform *ϕ*_SiO_2__, of approximately 0.2 (Fig. 4b). This uniformity persists throughout the rest of the sample, as evident from orthogonal 2D cross-sections (Fig. 4c, d, Fig. S2a and Video S3, ESI†). Note that a minor increase in *ϕ*_SiO_2__ is visible at the top, and a more substantial increase near the bottom. We attribute the latter deviation to interference between the silicon and silica signals, as mentioned before. To obtain accurate measurements, we exclude the substrate signal at the bottom and the minor air signal at the top, focusing only on the data that represent the dried film to calculate the average *ϕ*_SiO_2__ along the *z* direction (Fig. 4e). This analysis highlights the remarkable homogeneity of the sample, with *ϕ*_SiO_2__ remaining consistent throughout the entire sample. The red dashed line represents the expected *ϕ*_SiO_2__ = 0.18 if the latex and silica would be randomly mixed. Indeed, the computed *ϕ*_SiO_2__ closely matches this value, except for a slight increase near the top and a decrease in the middle, indicating minor silica enrichment at the air–film interface.

In the film dried at 70 °C, the enrichment of silica at the top appears significantly higher, with certain areas exhibiting remarkably high *ϕ*_SiO_2__ up to 0.9 (Fig. 4f). However, there are also regions at the top with *ϕ*_SiO_2__ ranging from 0.20 to 0.25, indicating considerable heterogeneity in this area. These variations are similarly visible in the 2D cross-sections, where high *ϕ*_SiO_2__ regions are concentrated at the top of the film, featuring distinct variations along the *x* direction (Fig. 4g, h, Fig. S2b and Video S4, ESI†). We note that in cracked regions, the determination of *ϕ*_SiO_2__ is inaccurate because the Raman signal is unclear, resulting in unrealistic silica intensity ratios in these areas. Nevertheless, a significant enrichment of silica near the air–film interface is still evident. In the vicinity of this interface, the laterally average *ϕ*_SiO_2__ is 0.45, well above the expected random *ϕ*_SiO_2__ of 0.18 (Fig. 4i). As we move deeper into the film (around *z* ≈ 40 μm), the volume fraction declines below 0.18. Hence, by converting the CRM signals to volume fractions, we not only identify areas of enrichment but also quantify the absolute extent of this enrichment.

### 3.2 Cross-sectional Raman microscopy

**Fig. 5:**
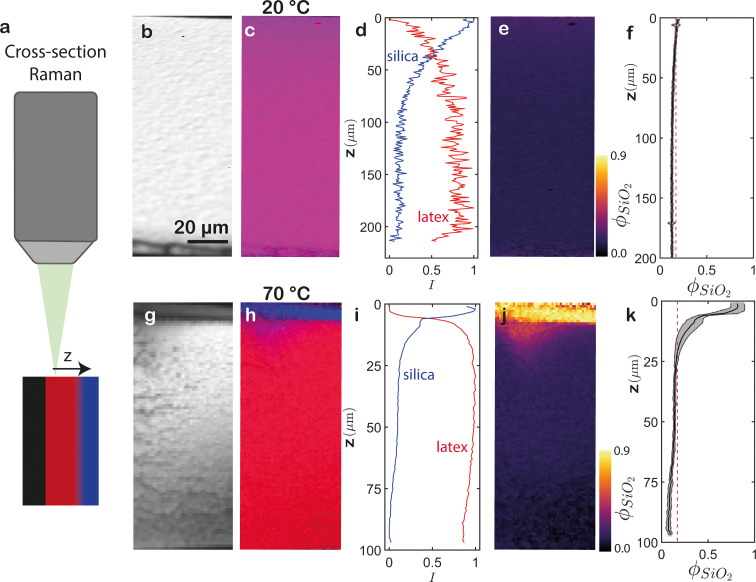
Raman microsopy of cross-sections of latex-silica blends dried at (b)–(f) 20 °C and (g)–(k) 70 °C. (a) Schematic of the experimental set-up. (b), (g) Integrated total Raman signal. (c), (h) Overlay of the integrated latex signal (red) and silica signal (blue). (d), (i) Laterally averaged and normalized intensities. These intensities are normalized by their respective highest and lowest values for standardization purposes. In (d), spatial variations consequently appear amplified. (e), (j) *ϕ*_SiO_2__ derived by converting the maps in (c) and (h) using the calibration curve. The scale bar in (b) applies to the *x* direction in all cross-sections. The *z* scale is given in (f) and (k). (f), (k) Laterally averaged *ϕ*_SiO_2__. The grey areas represent the standard deviation. The red dashed lines indicate the expected random *ϕ*_SiO_2__. These XRM measurements demonstrate superior resolution to CRM for both superficial and deeper layers.

Generating cross-sections of samples helps to overcome challenges posed by non-transparent regions, revealing otherwise obscured deeper layers and effectively eliminating other scattering-related artefacts. We obtain a flake of the film by dispersing the samples multiple times in liquid nitrogen, which causes cracking of the sample and flakes that detach from the substrate. On the same confocal Raman microscope as above, we can mount this flake at a 90° angle with the cross-section facing upwards, to achieve depth-resolved imaging in the lateral direction (indicated by *z* in Fig. 5a). Raman spectra can *e.g.* be acquired every μm^2^, and analyzed in a similar manner as the CRM data. However, XRM data require correction of the focus, since cross-sections are typically not perfectly flat. We do this by normalizing every collected Raman spectrum with the total integral of the intensities. Exemplary results for samples dried at 20 °C and 70 °C are shown in Fig. 5b and g, respectively. We use the corrected spectra to determine the integral of the latex and silica signals. In agreement with earlier conclusions, the cross-section of the film dried at 20 °C displays a uniform composition (Fig. 5c). In the signal obtained from the averaged latex and silica signals, a pronounced increase in the silica signal is observed close to the air interface (Fig. 5d). It is important to highlight that this signal is normalized based on the lowest and highest number of latex and silica signals acquired. As a result, the intensity differences are amplified. By utilizing the calibration curve described earlier, we mitigate the impact of these intensity differences and convert the pixel values into localized silica volume fractions. Similar to CRM, we observe a homogeneous *ϕ*_SiO_2__ that closely matches the expected random distribution throughout the entire sample (Fig. 5e and f). However, the erroneous substrate-silica cross-talk observed at the bottom of the CRM profiles (Fig. 4c–e) is absent from the XRM profiles. We note that the latter are also devoid of cracks; this is not an imaging flaw, but due to the sample preparation procedure and stochastic nature of fracture.

XRM of the 70 °C film corroborates the previously found stratification pattern in the CRM measurements, showing a noticeable concentration of silica near the air interface (Fig. 5h–i). Interestingly, the silica layer appears much sharper, both in width and in magnitude: the silica volume fraction is ∼0.85 ± 0.05 across a thickness of ∼5 μm (compare Fig. 4g–i and 5j–k). The disparity between these observations can be attributed to several factors. Firstly, the limited scope of the XRM analysis, which only covers one lateral dimension of the sample, fails to capture all lateral heterogeneities when compared to the 3D CRM measurements. Secondly, the nature of the CRM measurements introduces another source of differentiation: the refractive index mismatch at the air–film interface results in a displacement mismatch between the objective and the optically probed distance, causing a convolution effect in the *z* direction. As a consequence, the stratification appears less pronounced in the CRM data since multiple layers are sampled simultaneously. In contrast, the XRM data exhibit sharp stratification that is closer to reality.^33^ Thirdly, this signal blurring, in combination with scattering, leads to discrepancies deep inside the films. While the XRM profile shows a depletion of silica near the substrate (Fig. 5k), the CRM profile suggests a constant and even slightly rising *ϕ*_SiO_2__ (Fig. 4i). The former measurement is likely more accurate.

Our data show that, overall, CRM and XRM yield essentially the same information. CRM has the advantage of being direct and non-destructive with access to a third dimension. Nevertheless, as depth increases, the signal progressively convolutes, leading to less accurate information in deeper layers. In contrast, XRM offers superior depth resolution, enabling researchers to correctly analyze sharp features in the film, as well as (partially) opaque samples.

### 3.3 SEM–EDX imaging

Both CRM and XRM rely on unique variations in Raman scattering of the constituents to interpret the film composition. However, in some cases, these spectra may not exhibit substantial variations. In such scenarios, the components may still contain distinct elements, making elemental analysis *via* EDX spectroscopy a suitable means to determine spatial distributions. In this study, we use EDX in conjunction with SEM on similar cross-sections as above, to enable a comparison with the CRM and XRM data. As a proxy for the latex concentration we use the carbon (C) signal, and as a proxy for the silica concentration we use the silicon (Si) signal.

**Fig. 6:**
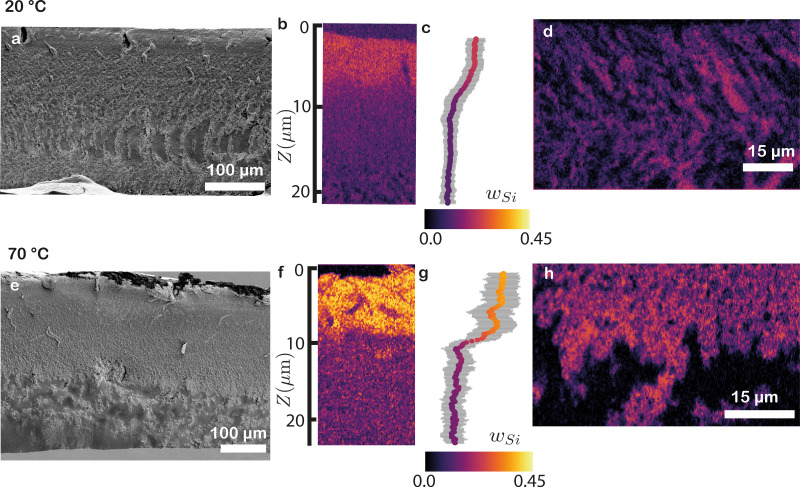
SEM and EDX imaging of cross-sections of latex-silica blends dried at (a)–(d) 20 °C and (e)–(h) 70 °C. (a), (e) SEM images of the full cross-sections. (b), (f) Silicon weight fractions of the top of the cross-sections determined using EDX. The colour coding is given in (c) and (g), along with the laterally averaged weight fractions. The grey area indicates the standard deviation. A clear surface excess of silica is observed for the 70 °C film, and only a minor excess for the 20 °C film. (d), (h) Silicon weight fractions near the bottom of the cross-sections, revealing pockets with alternating high and low silicon content, indicative of micro-segregation between the silica and latex. The colour coding is given in (c) and (g). These data show that SEM–EDX allows the quantitative mapping of different types of demixing.

SEM images of the 20 °C film illustrate a predominantly uniform film in the top half (Fig. 6a and Fig. S5a, ESI†). However, when we measure the elemental composition of a magnification of the top using EDX, we find a small increase in silicon content in a surface layer of ∼8 μm thick (Fig. 6b and Fig. S3 and S4a–d, ESI†). Taking the lateral average yields a silicon weight fraction (*w*_Si_) of 0.20, corresponding to *ϕ*_SiO_2__ = 0.22 (Fig. 6c). This value is slightly higher than the *ϕ*_SiO_2__ = 0.18 values found using CRM and XRM (Fig. 4c–e and 5e, f). As we delve deeper towards the middle portion of the film, morphologically heterogeneous structures become more apparent (Fig. 6a). Elemental analysis of these deeper layers shows that the structures consist of alternating patches with high and low *w*_Si_ (Fig. 6d and Fig. S4e–h, ESI†). The presence of this type of segregation remained concealed in both CRM and XRM analyses. Clearly, SEM–EDX measurements offer enhanced resolution capabilities, enabling the detection of even smaller heterogeneities.

At the top of the 70 °C film, a corrugated structure is visible (Fig. 6e). Further magnification of this region reveals numerous small silica particles (Fig. S5b and c, ESI†). This observation is supported by a significant increase in *w*_Si_ at the top of the film, reaching 0.4, which corresponds to *ϕ*_SiO_2__ ≈ 0.75 (Fig. 6f, g and Fig. S3i–l, ESI†). This pronounced segregation aligns well with the measurements obtained through CRM and XRM (Fig. 4g–i and 5j, k). We hypothesize that the corrugations are the result of excess of silica in the upper section of the film, as these rigid particles do not deform during the drying process, preventing relaxation of the high drying stresses and possibly leading to crack formation in the top layer. In the middle portion of the film, uneven structures emerge, while the lower region consists mostly of well-coalesced latex (Fig. 6e). This nearly pure latex layer agrees with our XRM measurements, which detected minimal to no silica at the bottom (Fig. 5e and f). This transition is also evident in the EDX data, where heterogeneous patches are observed in the middle, similar to the 20 °C sample, while the latex signal dominates at the bottom (Fig. 6h, see Fig. S3m–p for the full SEM–EDX analysis, ESI†).

Higher-magnification SEM images of these regions clearly show silica particles surrounded by well-coalesced latex particles (Fig. S5d and e, ESI†), indicative of significant demixing, resulting not only in vertical stratification but also in micro-segregation throughout (the middle region of) the films. To further investigate this behaviour, we create mixtures with different volume fractions of the two components in water (Fig. S6, ESI†). We find that, if *ϕ*_SiO_2__ exceeds 0.07, the mixture starts to phase separate. Although our initial silica volume fractions are below this volume fraction, during the evaporation process the concentration increases and eventually reaches this critical value, leading to local phase separation of the two components. As the sample continues to dry, the formed phase domains will at some moment be dynamically arrested and/or kinetically trapped.

These findings demonstrate how EDX imaging enables detailed elemental analysis of a sample, offering insights into the composition across various depths. Combining EDX with SEM allows for the correlation of structural variations with component distributions. Notably, SEM–EDX boasts superior resolution when compared to CRM and XRM, facilitating the identification of compositional differences in deeper regions and the unveiling of micro-segregation of different components.

## 4 Conclusions

In this study, we have compared three quantitative imaging techniques to gain detailed insights into the composition of a binary silica-latex film obtained by drying: confocal Raman microscopy, cross-sectional Raman microscopy, and energy dispersive X-ray spectroscopy. These methods do not require chemical labels or structural variations to create imaging contrast, but only spectral distinguishability of the components, making them well suited for characterizing complex coating systems. We have shown step by step how to apply these methods and optimize the acquired data. CRM has the main advantage of allowing reconstruction of a 3D map of the pristine sample, revealing vertical stratification in one go. XRM and EDX require fractured films, yet enable more highly-resolved interrogation of the entire thickness, identifying sharp transitions, from surface-segregated layers to the substrate. The integration of EDX with scanning electron microscopy further enables correlation of compositional variations with structural features at a submicrometric level, uncovering subtle heterogeneities in all directions.

CRM and XRM can be applied to a diverse range of systems, with the main prerequisite being that the Raman spectra of the individual components exhibit distinguishable differences. We note that although CRM allows 3D mapping, the Raman signal weakens with increasing depth, resulting in reduced resolution. This effect is even more pronounced in non-transparent samples, where only a thin surface layer can be measured accurately. Regions with cracks, cavities or other strongly scattering objects should therefore be interpreted with caution. In those cases, XRM is the preferred method. SEM–EDX is applicable to all systems whose components consist of distinct elements. While its spatially resolving power surpasses that of Raman microscopy, it is the most costly and resource-intensive technique.

A limitation of the described methodologies is the substantial time investment, particularly for generating high-resolution 3D CRM images. This could easily be improved by omitting one spatial axis or by sacrificing spatial resolution. The enhanced time resolution might even enable the observation of demixing dynamics during drying. However, several factors must be considered to achieve such measurements successfully. Notably, water exhibits its own Raman signal that could potentially interfere with other signals. Additionally, due to the dispersion of latex particles in the water phase, light scattering becomes a challenge, making it difficult to obtain accurate measurements at greater depths within the sample. Nevertheless, it might be feasible to conduct decent Raman measurements close to the air–water interface up to several tens μm into the drying film, allowing for time-resolved visualization of segregation processes.

In this paper, we have just scratched the surface of the potential applications for characterizing multi-component films. We hope that our described methodologies will inspire and encourage other researchers to adopt them in their own investigations of complex systems. Compositional heterogeneities in films are often overlooked, yet they can substantially impact material properties, both positively and negatively. Visualizing such patterns is the first step towards unlocking a deeper understanding of their significance, ultimately enabling the development of products with enhanced properties and performance.

## Author contributions

E. H., J. V. D. G., J. S. and H. M. V. D. K. conceived the study. E. H. and M. D. synthesized and characterized the latex colloids and performed the experiments and data analysis. All authors discussed the data and their interpretation and co-wrote the manuscript.

## Data and code availability

The raw data and code that support this study are available upon reasonable request.

## Conflicts of interest

There are no conflicts to declare.

## Supplementary Material

SM-019-D3SM01212C-s001

SM-019-D3SM01212C-s002

SM-019-D3SM01212C-s003

SM-019-D3SM01212C-s004

SM-019-D3SM01212C-s005
