# Supplementary material for: Quantitative imaging methods for heterogeneous multi-component films
Source: Soft Matter. 2023 Nov 6;19(45):8871–81. doi: 10.1039/d3sm01212c (PMC10663990; doi:10.1039/d3sm01212c)
Supplement: SM-019-D3SM01212C-s001 [file SM-019-D3SM01212C-s001.pdf]

Electronic Supplementary Material (ESI) for Soft Matter.
This journal is © The Royal Society of Chemistry 2023

Supplementary information for 'Quantitative imaging methods for heterogeneous multi-component films'

Ellard Hooiveld, Maarten Dols, Joris Sprakel, Jasper van der Gucht, Hanne M. van der Kooij

October 18, 2023

1 Supporting videos

Video S1
3D volume view of the integrated confocal Raman signals of the film dried at 20 °C, rotated over the z axis. The latex, silica and the silicon substrate signals are shown in red, blue and green respectively. Note that these colours are overlaid, leading to mixed colours. The scales are the same as in Fig. S1a. At the bottom, the silica signal seems to increase drastically, but this is an artefact related to the oxidation of the silicon substrate. This video shows a 3D representation of the data presented in Fig. 3a–e.

Video S2
3D volume view of the integrated confocal Raman signals of the film dried at 70 °C, rotated over the z axis. The latex, silica and the silicon substrate signals are shown in red, blue and green respectively. Note that these colours are overlaid, leading to mixed colours. The scales are the same as in Fig. S1b. This video shows a 3D representation of the data presented in Fig. 3f–j.

Video S3
3D volume view of ϕ_{SiO_2} of the film dried at 20 °C, rotated over the z axis. These values are derived from the CRM data in Video S1. The colour coding scale and the scale bars are the same as in Fig. S2a. At the bottom, ϕ_{SiO_2} seems to increase drastically, but this is an artefact related to the oxidation of the silicon substrate. This video shows a 3D representation of the data presented in Fig 4b–e.

Video S4
3D volume view of ϕ_{SiO_2} of the film dried at 70 °C, rotated over the z axis. These values are derived from the CRM data in Video S2. The colour coding scale and the scale bars are the same as in Fig. S2b. This video shows a 3D representation of the data presented in Fig. 4f–i.

2 Supporting figures

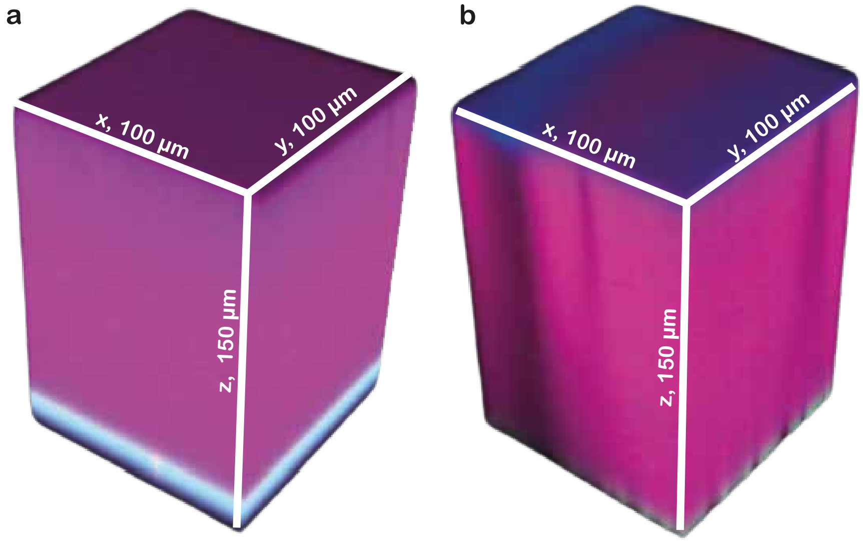

Fig. S1. 3D volume view of the integrated confocal Raman signals for the sample dried at (a) 20 °C and (b) 70 °C. The latex, silica and the silicon substrate signals are shown in red, blue and green respectively. At the bottom of (a), the silica signal seems to drastically increase, but this is an artefact related to the oxidation of the silicon substrate. For both films, we are able to capture the Raman signals in x y an z throughout the whole depth, and identify clear differences between a homogeneous and heterogeneous composition distribution. The signal is progressively more distorted in the deeper layers, due to scattering by refractive index mismatches, this can be resolved by cross-section Raman microscopy as shown in Fig. 5. For the 3D views from different angels, see Video S1 and Video S2. This figure shows a 3D representation of the data presented in Fig. 3.

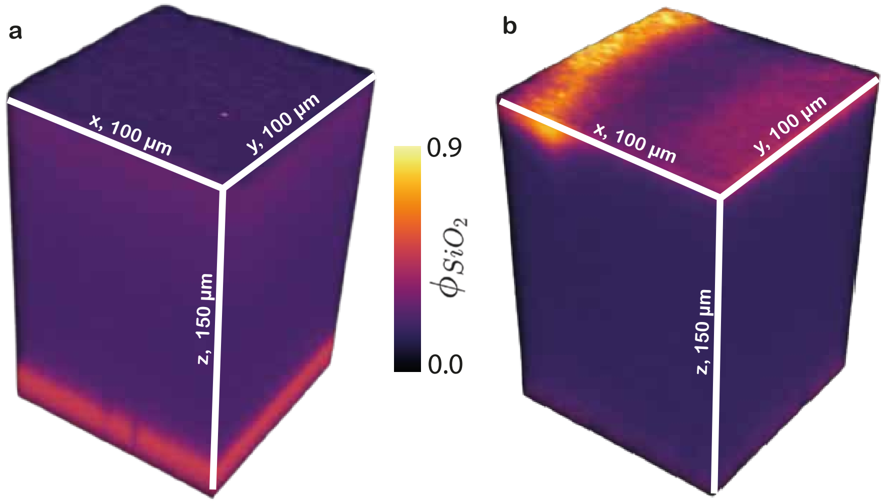

Fig. S2. 3D volume view of ϕ_{SiO_2} for (a) 20 °C and (b) 70 °C. The colour coding scales with the silica volume fraction indicated on the colour bar. At the bottom of (a), the silica signal seems to drastically increase, but this is an artefact related to the oxidation of the silicon substrate. The data from Fig. S1 is converted to quantitatively to ϕ_{SiO_2} and shown here. For the 3D views from different angels, see Video S3 and Video S4. This figure shows a 3D representation of the data presented in Fig. 4.

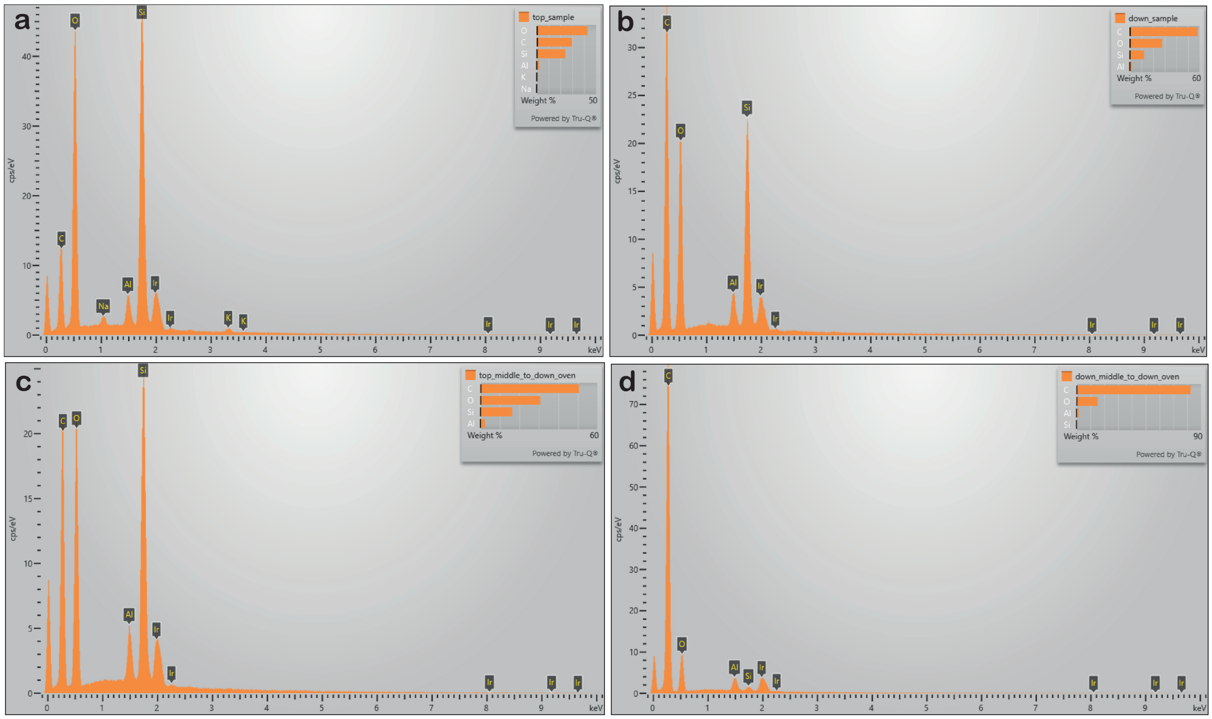

Fig. S3. Typical EDX elemental analysis spectra of different areas in the cross-section of the film dried at 70 °C: (a) close to the top, (b) just below the top, (c) in the middle, and (d) at the bottom. The EDX spectra correspond to the areas indicated by white squares in Fig. S4i-l for a (upper) and b (lower) and figure S4m-p for c (upper) and d (lower). The insets show the corresponding weight fractions of the detected elements. Traces of Al (mounting substrate), Na, K and Ir (sputter-coated element) are excluded from the final analysis of w_{Si} in Fig. 6. The silicon and oxygen signals can be found at the same positions, revealing the silica nanoparticles (which are mainly composed out of Si and O). The carbon signal mainly represents the latex phase.

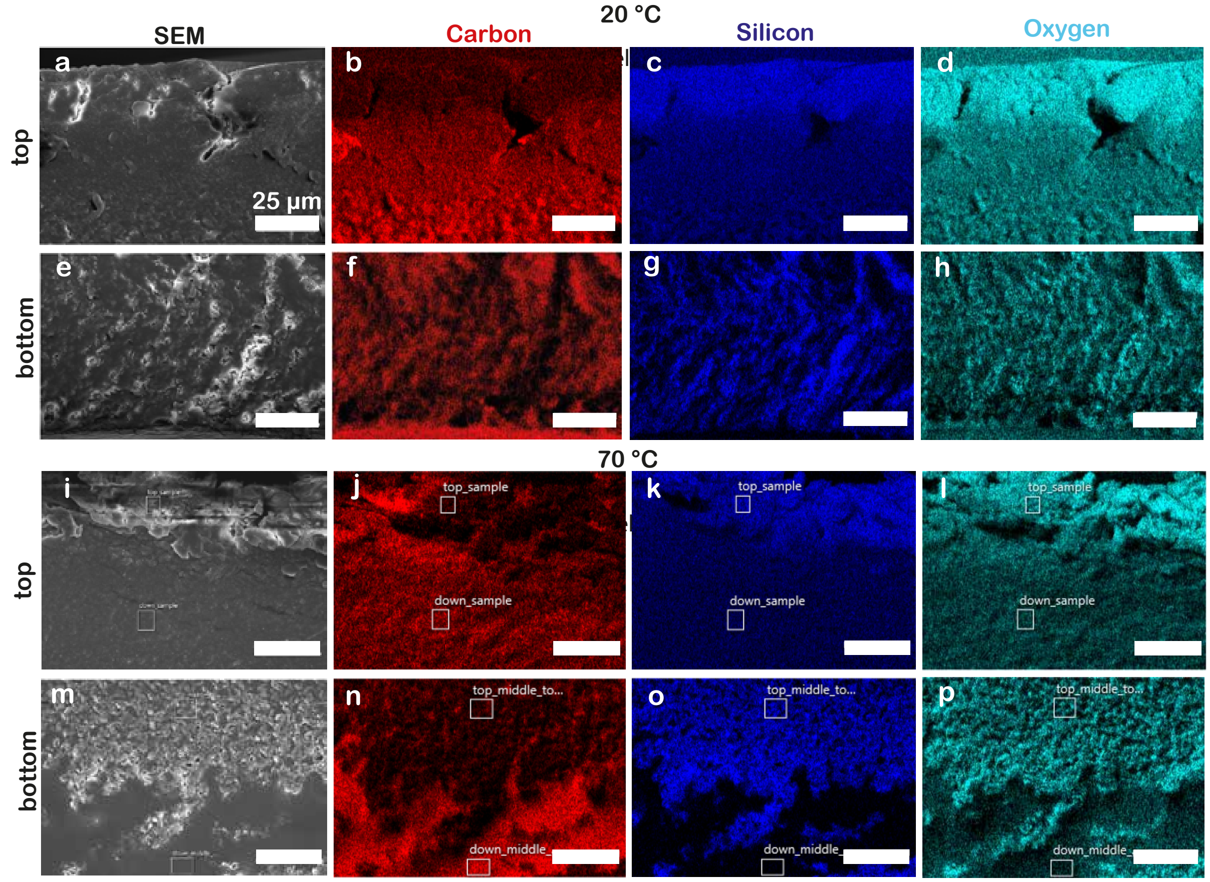

Fig. S4. SEM and EDX images. (a-d) Top and (e-h) bottom of the sample dried at 20 °C. (i-l) top and (m-p) bottom of the sample dried at 70 °C. The scale bars represent 25 μm. The silicon and oxygen signals show a clear overlap, both peaking near the top, while their magnitude negatively correlates with the carbon signal. These findings indicate enrichment of silica nanoparticles and depletion of latex near the top. The white squares in (i-p) represent the areas where different EDX spectra were created, which can be found in Fig. S3. These images show the full data of the SEM–EDX measurements presented in Fig. 6

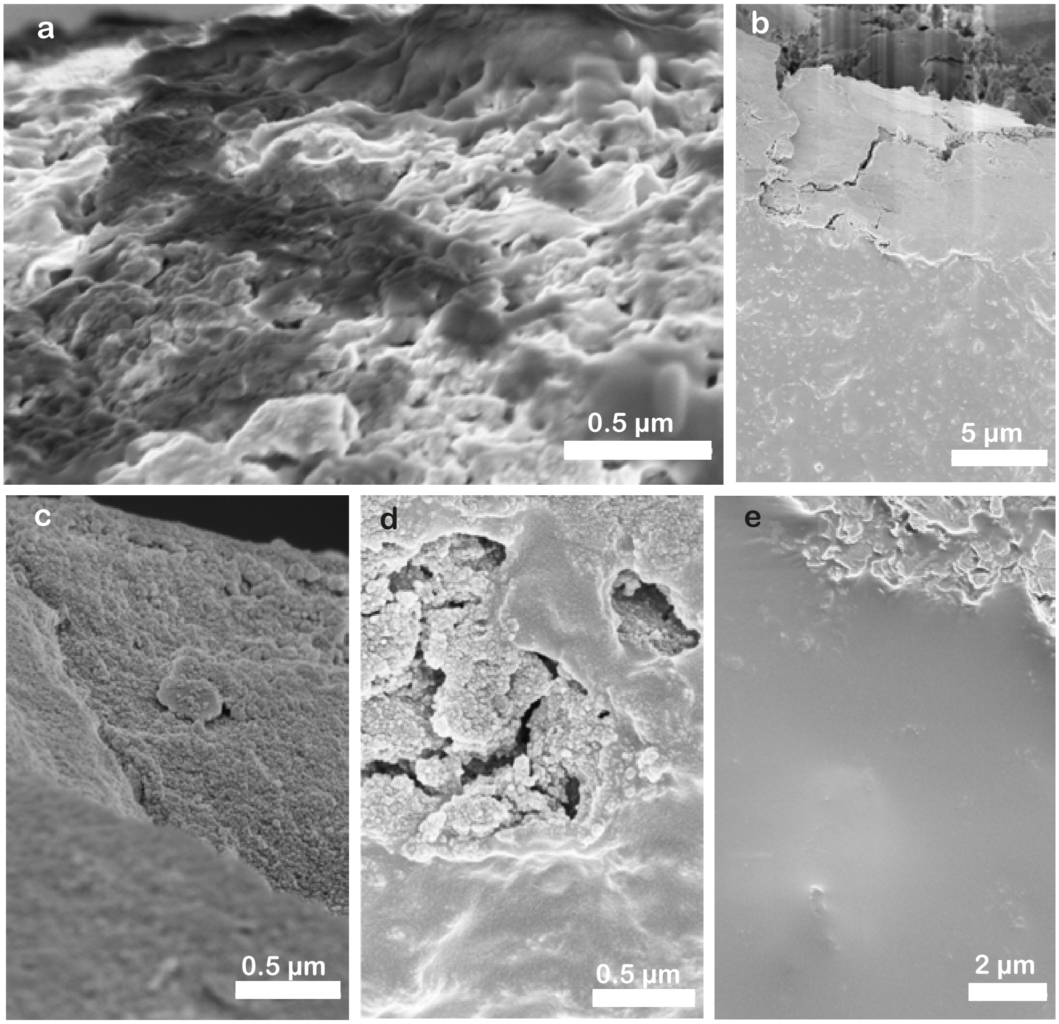

Fig. S5. SEM images of the top of the film dried at (a) 20 °C and (b) 70 °C. (c) Magnification of the top of the film dried at 70 °C. We clearly observe a large amount of silica particles, in contrast to the top of the film dried at 20 °C. Magnification of a patch with mainly (d) silica and (e) latex at z ≈ 100 μm of the 70 °C film. This reveals micro-segregation of the two components in the final dried binary film.

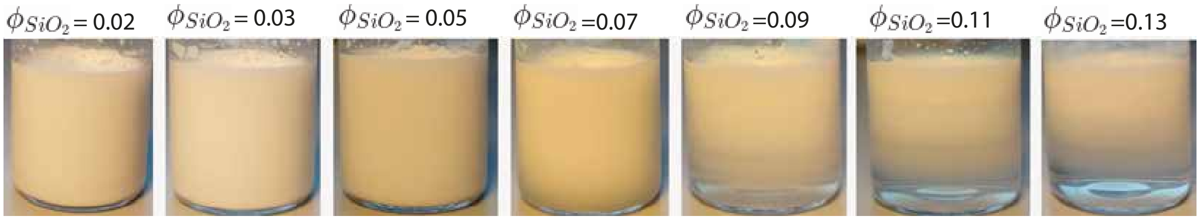

Fig. S6. Blends of silica and latex particles in water after two months of equilibration, highlighting phase separation beyond ϕ_{SiO_2}= 0.07. (At that point the phase separation is very minor, yet it becomes more pronounced from ϕ_{SiO_2}= 0.09 onward). Upon phase separation, the white turbid phase contains mostly large and scattering latex particles, while the transparent phase contains mostly silica particles that hardly scatter the light. With this experiment we confirm that phase separation of the components during drying may indeed occur once a critical volume fraction is reached, and concurrently may induce extra heterogeneities in the final dried film.
